# Explicit Kinetic Heterogeneity: Mathematical Models for Interpretation of Deuterium Labeling of Heterogeneous Cell Populations

**DOI:** 10.1371/journal.pcbi.1000666

**Published:** 2010-02-05

**Authors:** Vitaly V. Ganusov, José A. M. Borghans, Rob J. De Boer

**Affiliations:** 1 Theoretical Biology, Utrecht University, Utrecht, The Netherlands; 2 Institute of Biophysics, Krasnoyarsk, Russia; 3 University Medical Center Utrecht, Utrecht, The Netherlands; University of Oxford, United Kingdom

## Abstract

Estimation of division and death rates of lymphocytes in different conditions is vital for quantitative understanding of the immune system. Deuterium, in the form of deuterated glucose or heavy water, can be used to measure rates of proliferation and death of lymphocytes in vivo. Inferring these rates from labeling and delabeling curves has been subject to considerable debate with different groups suggesting different mathematical models for that purpose. We show that the three most common models, which are based on quite different biological assumptions, actually predict mathematically identical labeling curves with one parameter for the exponential up and down slope, and one parameter defining the maximum labeling level. By extending these previous models, we here propose a novel approach for the analysis of data from deuterium labeling experiments. We construct a model of “kinetic heterogeneity” in which the total cell population consists of many sub-populations with different rates of cell turnover. In this model, for a given distribution of the rates of turnover, the predicted fraction of labeled DNA accumulated and lost can be calculated. Our model reproduces several previously made experimental observations, such as a negative correlation between the length of the labeling period and the rate at which labeled DNA is lost after label cessation. We demonstrate the reliability of the new explicit kinetic heterogeneity model by applying it to artificially generated datasets, and illustrate its usefulness by fitting experimental data. In contrast to previous models, the explicit kinetic heterogeneity model 1) provides a novel way of interpreting labeling data; 2) allows for a non-exponential loss of labeled cells during delabeling, and 3) can be used to describe data with variable labeling length.

## Author Summary

Understanding of cellular processes is impossible without quantitative estimates of how quickly cells in an organism divide and die. The most widely used approach to measure rates of cell turnover in humans is by labeling dividing cells with deuterium given in the form of deuterated glucose or heavy water. Surprisingly, quantitative estimates of the rates of cell turnover obtained from accumulation and decay of the labeled nucleotides in the cell population varied between different studies. We demonstrate that these differences were not likely to arise because of different mathematical models used in data fitting, since the previously used models have an identical mathematical structure. We extend these previous models to allow for cell populations with different rates of turnover and show how such a new explicit kinetic heterogeneity model can be applied to simulated and experimental data. The new model opens a new way of interpreting data from deuterium labeling experiments and will likely lead to new insights into how infections and/or treatments affect cell turnover in humans.

## Introduction

There is little consensus about the expected life spans of lymphocyte populations in health and disease. Labeling the DNA of dividing cells with deuterium has proved to be one of the most reliable and feasible ways to study the population dynamics of lymphocytes in healthy human volunteers and in patients [1],[2],[3]. Deuterium, in the form of deuterated glucose or heavy water, is used to measure the rate at which cells are dividing *in vivo*, without the need to interfere with these cellular kinetics. Deuterium is incorporated into newly synthesized DNA via the *de novo* pathway [4], and enrichment of deuterium (over hydrogen) in the DNA of cells is therefore related to cell division. During label administration, the fraction of deuterium-labeled nucleotides increases over time, and after label withdrawal, the fraction generally declines over time [2],[3]. Labeling DNA with deuterium in humans has a number of clear advantages over other labeling techniques such as with BrdU, including the absence of toxicity, the fact that the rate of incorporation of deuterium into the DNA is independent of the amount of nucleotides present, and a simpler mathematical interpretation of the data [5],[6],[4]. Several mathematical models have been proposed for estimation of cellular turnover rates from labeling data [1],[2],[7],[8],[9],[10].

In their study on deuterium labeling, Mohri et al. [2] found that the estimated rate of cell proliferation was typically smaller than the rate of cell death. Because the cell population under investigation was in steady state, the extra death must be compensated by a source of cells, for example from the thymus. This interpretation was challenged by the work of Asquith et al. [9], which pointed out that estimated proliferation and death rates do not have to be equal if the population is kinetically heterogeneous (i.e., different cells in the population divide and die at different rates). Because the labeled population preferentially contains cells that proliferate (and die) relatively rapidly, the estimated rate of cell death is in fact expected to be higher than the average proliferation rate [9].

Here we extend these studies and propose an alternative approach to estimate the rates of lymphocyte proliferation and death from deuterium labeling experiments. First, we show that the three most commonly used mathematical models lead to identical estimates of the average rate of cell turnover and only differ in their biological interpretation of the model parameters. Second, we formulate a novel mathematical model which explicitly takes into account kinetic heterogeneity of lymphocyte populations, and show how lymphocyte turnover rates can be calculated using this model. Several previously made experimental observations arise naturally from the new model. For example, we find that the rate of label loss during delabeling generally exceeds the rate of label accumulation during the labeling phase. Our model also explains the dependence of the rate at which labeled DNA is lost after label withdrawal on the duration of the labeling period [9]. As a proof of principle, we demonstrate that the newly developed model can fit artificially generated data, and correctly returns their underlying kinetic parameters. We also illustrate the usefulness of the new model by fitting it to several experimental datasets. The novel explicit kinetic heterogeneity model may offer alternative interpretations of how infections or treatments affect the turnover of human lymphocytes *in vivo*.

## Results

### Previous models

Although different models have been proposed for interpretation of deuterium labeling data [2],[9] and are being debated in the literature, they are in fact mathematically identical, i.e., they predict mathematically identical labeling curves with one parameter for the exponential up and down slope, and one parameter defining the maximum labeling level. Following De Boer et al. [11], consider a cell population consisting of a fraction 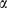 of cells with average turnover rate 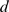 (i.e., an expected life span of 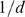 days), and a fraction 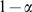 of cells that do not turnover at all on the time scale of the experiment. During the labeling phase, consider the fraction of unlabeled DNA 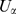 in the sub-population with death rate 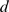. Because DNA is only lost by cell death, 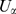 changes according to:
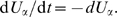
During the delabeling phase the fraction of labeled DNA in that same population (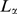) is described by:
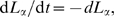
because labeled DNA can only be lost by cell death. Since 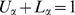, the fraction of labeled DNA in the whole population 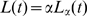 is described by:
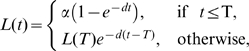
(1)where 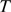 is the duration of the labeling period. Given that only a fraction 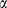 of all cells in the population are turning over (or dying) at rate 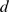, the average turnover rate of the whole population is 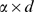 [11]. Importantly, this approach does not require us to describe how new cells are formed, i.e., they could be generated by the thymus and/or by proliferation. As in our previous work [11], this model assumes that the source produces cells with labeled DNA during the labeling phase, and cells with unlabeled DNA during the delabeling phase. This is in contrast with the model by Mohri et al. [2] which allowed the source to produce both cells with labeled and unlabeled DNA during both the labeling and the delabeling period. The reason for this simplification is that the model by Mohri et al. [2] was over-parameterized, i.e., the different source constants cannot be reliably determined from most labeling data (see [2] and results not shown). Moreover, the simpler model with a source of cells with only labeled or unlabeled DNA, typically describes the data with similar quality as the more complicated models (e.g., [11]).

Because the fraction of labeled nucleotides cannot exceed one, there is always a trivial asymptote at 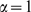. The explicit asymptote 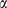 defined in the above model (and those discussed later) implies that even after infinite labeling, the fraction of labeled nucleotides will be saturated at a level 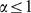, which could be due to the presence of non-dividing cells.

**Figure 1:**
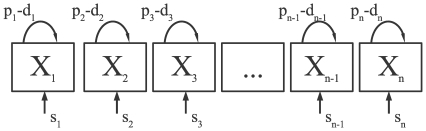
A cartoon of the model with explicit kinetic heterogeneity. In the model, the population of cells consists of 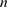 sub-populations with different rates of turnover. In the 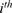 sub-population, there is a source of new cells that enter the cell population at rate 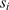 cells per day, cells divide at rate 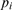 per day, and die at rate 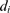 per day. To maintain the size of all sub-populations constant, 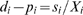 for every sub-population 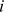, where 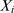 is the number of cells in the 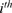 sub-population. In this model we assume that the source produces only labeled cells during the labeling phase, and delabeled cells during the unlabeling phase [11].

Extending the simple model given in Eqn. (1)by assuming 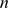 sub-populations with different rates of cell proliferation 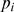 and death 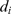, and possibly generation of new cells from a source 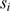 (Figure 1), the fraction of labeled nucleotides in the whole population at time 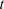 is given by:
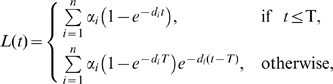
(2)where 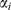 is the fraction of cells in population 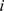 with death rate 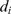, and 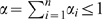 is the asymptote that would be approached if label would be administered indefinitely. The only requirement for the model defined by eqn. (2) is that cells within a given sub-population must have identical kinetic properties. For instance, in the absence of an acute infection, we expect that a clone of T cells with the same antigenic specificity may form a sub-population with identical kinetic properties (although there is no experimental evidence for that, see also Discussion section). In our model, new cells are produced by proliferation and from a source (Figure 1). For naive T cells, the source could represent production of cells by the thymus and for memory T cells the source could represent activation of resting cells [12],[11]. Even though the biological interpretation of the source may not always be clear, this forms no problem from a mathematical point of view, because the source term 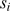 never enters the expression for the fraction of labeled DNA (see Eqn. (2)).

The “source” model that was previously proposed by Mohri et al. [2] considered one homogeneous cell population, but allowed for a source of unlabeled cells during the labeling phase, i.e., 
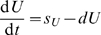
, which also gives rise to an asymptote 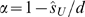, defining the fraction of cells that can maximally become labeled (here 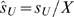 where 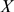 is the total number of cells in the population at equilibrium and 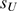 is the number of cells with unlabeled DNA coming from the source per day during the labeling phase [2]). Mathematically, the source model is therefore identical to eqn. (1). Similarly, in the kinetic heterogeneity model devised by Asquith et al. [9], 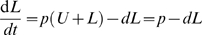 for the labeling phase and 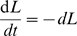 for the delabeling phase. Assuming 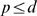 one again obtains Eqn. (1)with 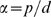. Therefore, all these models are mathematically identical and only differ in the biological interpretation of their model parameters (see also [13]). We propose to call all these models the “asymptote model”. Importantly, in all models the product 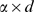 can be interpreted as the average rate of cell turnover of the population as a whole [11], and therefore, all three models, when fitted to data, will deliver identical estimates of the average turnover rate, which is the parameter of key interest. There is an important drawback of this approach, however. By interpreting labeling data only in terms of the average turnover rate one may not be able to explain why the average turnover rate is different, for example, between healthy controls and infected patients, and what the consequences of such a difference may be. One would need a particular biological model to explain such a difference. However, our results show that multiple models could be consistent with the labeling data and therefore, model specific predictions arising from labeling data alone may not be robust to changes in the model assumptions.

### Kinetic heterogeneity model with continuously distributed turnover rates

Because of its simplicity, the model given in eqn. (1) has two limitations. First, the asymptote level is a phenomenological parameter that depends on the length of the labeling period [9]. As a consequence, datasets with different labeling periods will likely give rise to different estimated asymptotes and different estimated average rates of cell turnover. Therefore strictly speaking, this model cannot be used to explain multiple datasets coming from the same experimental setup varying only in the length of the labeling period; the differences in the rate at which labeled DNA is lost would force either the asymptote or the estimated average turnover rate to be different for the different labeling periods (Den Braber et al. in preparation). Second, the model assumes that the increase in labeled DNA during the uplabeling phase, and the loss of labeled DNA during the delabeling phase can be described by single exponentials. This may be incorrect if cell populations with different turnover rates are labeled and subsequently lost.

**Figure 2:**
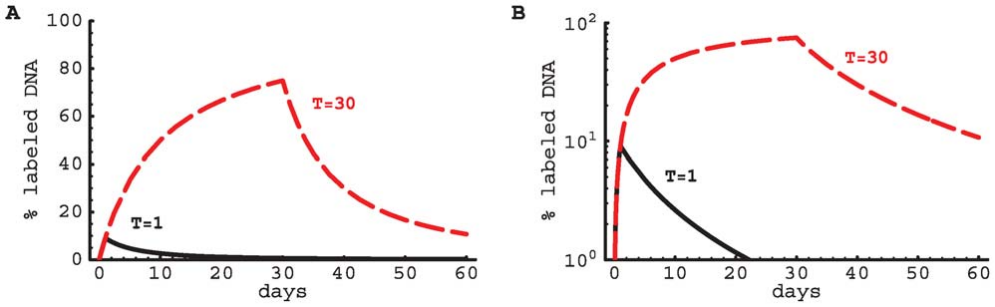
Model predictions for exponentially distributed turnover rates. We have plotted the changes in the fraction of labeled DNA according to the explicit kinetic heterogeneity model with exponentially distributed turnover rates (eqn. (5), mean 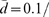
day). Predicted changes are shown for a short labeling period (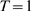 day, solid line) and a long labeling period (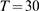 days, dashed line) on a linear (panel A) and a logarithmic (panel B) scale. The initial uplabeling rate is independent of the length of the labeling period and is given by 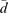. The initial rate of delabeling, in contrast, depends on the length of the labeling period and is approximately twice as fast in the case of short-term labeling as compared to long-term labeling.

Under very general assumptions, we have formulated an alternative model that does not make these *a priori* assumptions. In our new model, a cell population consists of 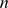 sub-populations each with different kinetic properties (see Figure 1 and eqn. (2)). If the number of sub-populations is large (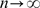), the sum in Eqn. (2) can be replaced by an integral. The fraction of labeled nucleotides in the population then becomes (see Text S1 for derivation)
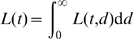
(3)where 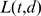 is given by Eqn. (1)where 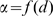 is the frequency distribution of turnover rates, and 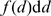 is the probability that a randomly chosen cell in the population belongs to a sub-population with a turnover rate in the range 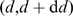. If the turnover rates in the population, 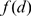, follow a gamma distribution, the change in the fraction of labeled DNA with time is given by:
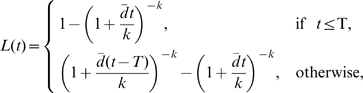
(4)where 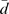 is the average rate of cell turnover in the population, 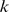 is the shape parameter of the gamma distribution, and 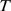 is the duration of the labeling period. For 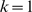, the gamma distribution becomes an exponential distribution, and the rate at which the fraction of labeled DNA changes is simply:
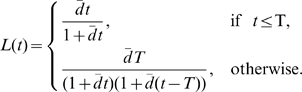
(5)This is an interesting model in which a single parameter 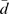 predicts both the rate of uplabeling and downlabeling, and in which there is no asymptote below 100% for the level of labeled DNA, i.e., under continuous label administration all cells in the population will become labeled (Figure 2). Moreover, this model predicts that the initial rate 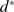 at which labeled DNA is lost after label cessation depends on the duration of the labeling period, 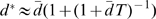 (see Text S1 for derivation). According to this model, short labeling experiments (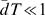, 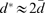) will lead to 2-fold faster initial rates of decline in the fraction of labeled nucleotides than longer labeling experiments (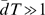, 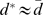).

**Figure 3:**
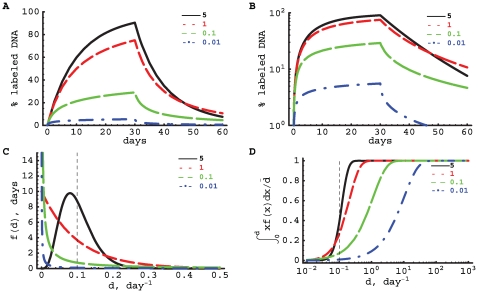
Model predictions for gamma-distributed turnover rates. We have plotted the changes in the fraction of labeled DNA according to the kinetic heterogeneity model with gamma-distributed turnover rates (eqn. (4)) with average turnover rate 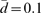/day on a linear (panel A) or logarithmic (panel B) scale. Predicted changes are shown for different values of the shape parameter 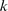. Larger values of 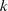 correspond to a more symmetric distribution (Panel C). For low values of the shape parameter 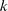, the loss of labeled DNA after label cessation is biphasic, which is most clearly visible on a logarithmic scale for 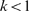 (panel B). This characteristic of the kinetic heterogeneity model differs from the Asymptote models which have a constant *per capita* rate at which labeled DNA is lost. Note that for shape parameters 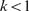, the distribution of turnover rates 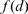 becomes extremely skewed with most cells undergoing hardly any division and relatively few cells undergoing extremely many rounds of division (panel C). Panel D gives the cumulative contribution of sub-populations with a particular turnover rate 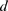 to the average rate of turnover of the population 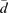. The vertical line shows the value of the average proliferation rate 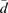. For high values of the shape parameter (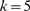), the cell sub-populations with turnover rates that are somewhat lower or higher than 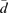 give the main contribution to the average turnover rate. In contrast, for low values of 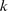 (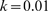), the major contribution to the average turnover rate comes from sub-populations with extremely rapid turnover rates (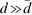); about 50% of the average turnover is due to a few sub-populations with turnover rates that exceed 10 per day, which is biologically unrealistic.

Solutions (4) and (5) predict that the initial rate of increase in the fraction of labeled DNA is the average rate of cell turnover 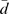 (see also Text S1). However, the increase in the fraction of labeled DNA does not appear to be exponential, as was implicitly assumed in the asymptote models discussed above. Similarly, during the delabeling period, the model predicts a non-exponential decline in the fraction of labeled DNA (Figures 2 and 3). In general, the initial rate of label loss during delabeling 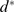 is given by:
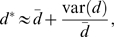
(6)where 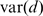 is the variance of the distribution of turnover rates in the population. In case when turnover rates follow a gamma distribution, the initial rate of loss of the label after short labeling periods depends on the shape parameter 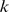 of the distribution, 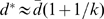, while it does not after long labeling periods (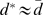). The rate of loss of labeled DNA slows down as less DNA remains labeled, which is most clearly seen when proliferation rates are distributed according to a very skewed gamma distribution (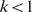, Figure 3). This is a natural property of the explicit kinetic heterogeneity model as loss of labeled DNA is reflecting the distribution of the turnover rates of the different sub-populations, with labeled DNA from the most rapidly turning over sub-populations being lost first (early fast decline) and labeled DNA from the other, more slowly turning over, populations being lost later (late slow decline).

To study the effect of the shape of the turnover rate distribution on the predicted labeling curve, we plotted the changes in the fraction of labeled DNA as predicted by the model (Figure 3A&B) with different gamma-distributed turnover rates (Figure 3C). When the gamma distribution is highly skewed (i.e., 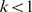), the majority of cell sub-populations have very low rates of cell turnover, and the average rate of cell turnover is dominated by a few sub-populations that turn over unrealistically fast. This is best illustrated by calculating the cumulative contribution of a sub-population with a particular rate of turnover to the average turnover rate of the population 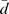 (Figure 3D):
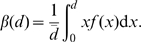
(7)


For large values of the shape parameter 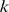 (e.g., 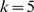), the sub-populations with turnover rates that are close to the average turnover rate 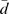, are the main contributors to the average rate of cell turnover (Figure 3D). When the gamma distribution is extremely skewed (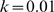), the rate of turnover of the sub-populations that contribute significantly to the average turnover rate is as high as 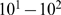 per day, which is biologically unrealistic. Therefore, the gamma distribution should be rejected whenever one estimates a high average turnover rate 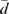 with a highly skewed gamma distribution (i.e., a low value of the shape parameter 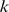). As a rule of thumb, 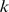 should be larger than 0.1 (Figure 3D); otherwise a relatively large fraction of sub-populations has unrealistically high turnover rates.

It is possible, however, that not all cells in the population are turning over. The models above can easily be extended to incorporate this possibility by allowing for the same asymptote as in eqn. (1). An example would be a labeling experiment in which slowly turning over naive T lymphocytes and more rapidly turning over memory lymphocytes are not separated [2]. If only a fraction 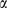 of cells have turnover rates that are distributed exponentially, and the other cells undergo negligible turnover on the time scale of the experiment, the change of the fraction of labeled nucleotides with time is given by:
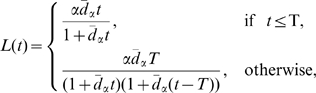
(8)where 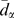 is the average of the exponentially distributed turnover rates, and the average rate of cell turnover in the whole population is 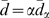.

It should be noted that the results of this section are applicable both to proliferating and non-proliferating lymphocytes, given the general structure of the cell population in the model (see Figure 1). As a downside of this, the model does not allow to estimate which fraction of labeling of lymphocytes is due to proliferation of precursors (e.g., thymocytes for naive T cells) or due to peripheral proliferation of the lymphocyte population itself. Additional experiments, such as thymectomy in case of studies of naive T-cell turnover, may allow to estimate the separate contribution of peripheral T-cell proliferation [14, Den Braber et al. submitted].

### Fitting artificial data to validate the model

Having analytical expressions for several kinetic heterogeneity models, we analyzed how well these models can recover the (known) average turnover parameter from simulated (artificial) datasets. Three models were used to generate artificial datasets: 1) the kinetic heterogeneity model with gamma-distributed rates of turnover (eqn. (4)), referred to as the “Gamma model”, 2) the kinetic heterogeneity model in which a fraction 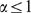 of cells have exponentially-distributed rates of turnover (eqn. (8)), referred to as the “Exponential model”, and 3) a “Two population model” (Eqn. (2) with 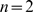, turnover rates 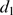 and 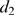, average turnover rate 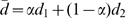, and 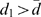). These datasets were subsequently fitted by the same three models as well as by the conventional Asymptote model (eqn. (1)).

**Figure 4:**
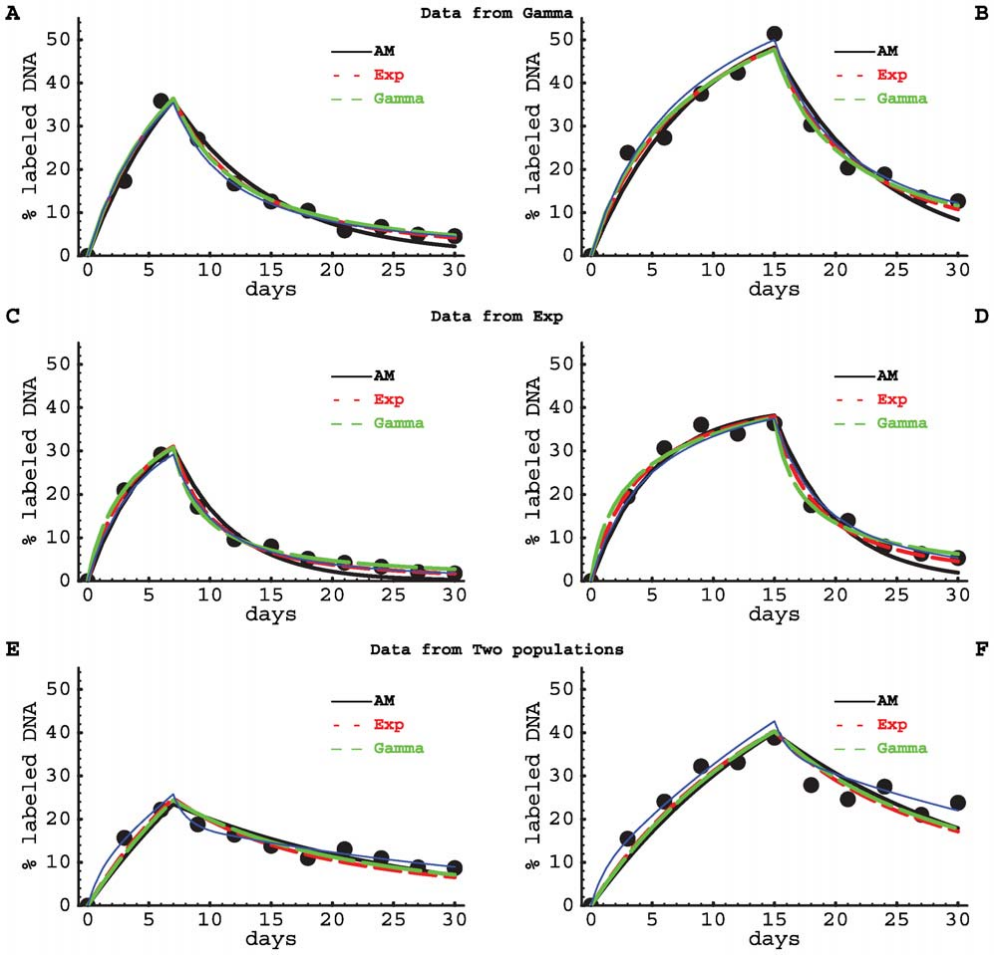
Fits of artificial (simulated) data with various models. We have fitted artificial data (black dots) with the Asymptote model (eqn. (1), solid black lines), the Exponential model, in which a fraction 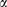 of the cells have exponentially distributed turnover rates (eqn. (8), small red dashed lines), and the Gamma model with gamma distributed turnover rates (eqn. (4), large green dashed lines). Data were generated using the Gamma model (panel A&B), the Exponential model (panel C&D) and the Two-populations model (panel E&F, Eqn. (2)), respectively. Thin blue lines show the exact curves of the models that were used to generate the data. The different models were fitted to 11 datapoints taken from these predicted curves after having added noise to these data points. Noise was added by a relative change of the predicted value with a normally distributed error (with standard deviation of the distribution 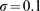). The models were fitted to data from artificial labeling experiments in which the label was administered for 7 (left panels) or 15 (right panels) days. Parameter estimates providing the best fit are shown in Table S1, and the corresponding estimates of the average rates of cell turnover 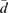 are shown in Figure 5. Parameters used to generate the data are also given in Table S1.

**Figure 5:**
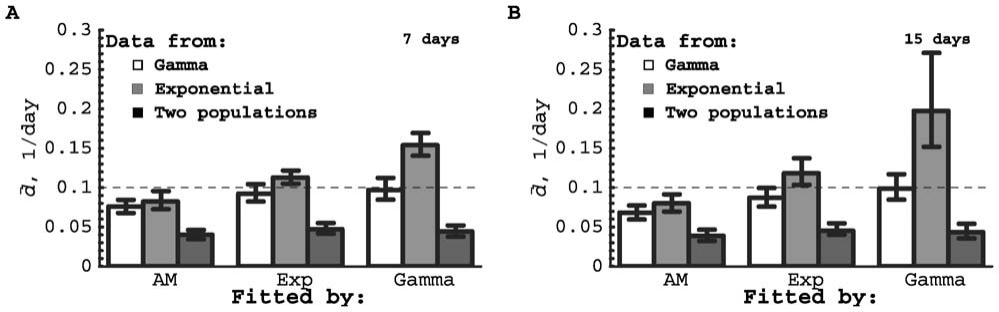
Average turnover rate estimated obtained by fitting mathematical models to simulated data. By fitting the artificial data described in the text, we estimated the average turnover rate using three models: the Asymptote model (eqn. (1)), the Exponential model (in which a fraction 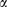 of cells have exponentially distributed turnover rates, eqn. (8)), and the Gamma model (with gamma-distributed turnover rates, eqn. (4)). Estimated mean values and 95% confidence intervals obtained by bootstrapping the residuals with 1000 simulations are shown. Data were generated using the Gamma model (empty bars), the Exponential model (gray bars) and the Two-populations model (black bars). Labeling periods were 7 (panel A) and 15 (panel B) days. Horizontal dashed lines denote the actual average rate of lymphocyte turnover in all data, 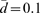/day. Note that in this example, the Asymptote model always underestimated the average rate of cell turnover, and that there is a systematic 2-fold underestimation of the average turnover by all models when the data from the Two-populations model were fitted. This is because all three models fail to describe the relatively rapid accumulation of the label at early time points (see Figure 4E–F).

Not surprisingly, the models delivered correct estimates for the average turnover parameter if a dataset was fitted with the model that was used to generate the data (Table S1 and Figures 4 and 5). All models described the data sets generated by the other models reasonably well (Figure 4), although some features in the data could not be reproduced. For example, the Asymptote model failed to describe the decreasing rate at which labeled DNA is lost over time, which is observed in the data generated by the Gamma and the Exponential models (see last data points in Figure 4). Some model fits delivered incorrect estimates for the average turnover rate if the data were generated using another model. For example, the Gamma model overestimated the average turnover rate when the data were generated using the Exponential model (up to 2-fold), and underestimated 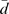 for data generated using the Two populations model (over 2-fold). This is most likely due to the strong constraint of the model that both uplabeling and delabeling curves have to be described with one mechanism, i.e., gamma-distributed turnover rates. On the other hand, the Asymptote model always underestimated the true average turnover rate (up to 2-fold for data generated by the Two-populations model; Table S1). It did perform somewhat better than the Gamma model as judged by the mean square distances, because the rate of uplabeling and downlabeling are relatively independent in the Asymptote model.

Given that natural lymphocyte populations are likely to contain resting sub-populations, some extent of saturation in the fraction of deuterium-labeled nucleotides is expected in almost any experimental dataset. In our artificial data, such an asymptote was imposed when using the Exponential model by letting only 50% of all cells to turn over (Figure 4 and Table S1). It is therefore not surprising that the Gamma model, which does not have an explicit asymptote in the uplabeling phase (see eqn. (4)), did not correctly estimate the average turnover rate for the data generated by the Exponential model (Figure 5). Extending the Gamma model to allow for an explicit asymptote during the labeling phase (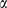) indeed improved the estimate of the average turnover rate 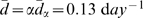 (with 95% CIs = 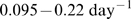 which includes the true average 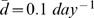), even though the estimated fraction of turning over cells 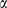 was not significantly different from 1 (i.e., an F-test would not reject a model with 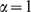; results not shown). This exercise illustrates that when fitting experimental data one should check whether allowing for an explicit asymptote in the uplabeling phase leads to different estimates of the average turnover rate. Interestingly, all models underestimated the average rate of cell turnover when the data were generated using the Two populations model. This is because the models did not reproduce the relatively rapid accumulation of the labeled DNA in the first days (Figure 4). Fitting the Two populations model to these data led to better estimates of the average turnover rate (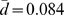 per day with 95% CIs = 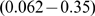 for 7 days of labeling, and 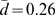 per day with 95% CIs = 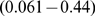 for 15 days of labeling, where the constant 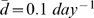 is contained within both ranges, results not shown).

Although stable isotope labeling seems to be the best tool at hand to estimate rates of lymphocyte turnover, a recent review [10] pointed out that estimated lymphocyte turnover rates differ consistently, depending on the labeling method used (heavy water or deuterated glucose), and the length of the labeling period. *A priori*, according to the Asymptote model that is generally used, the estimated average turnover rate should not depend on the length of the labeling period. Using our explicit kinetic heterogeneity models, we analyzed the influence of the length of the labeling period on the estimated average turnover rate. For all models, we found that the duration of labeling had little influence on the estimated average turnover rate (for the chosen labeling periods of 7 and 15 days, Table S1). This suggests that longer labeling periods will not necessarily result in lower estimates of the average cell turnover rate than shorter labeling periods.

An overall conclusion of this analysis is that without a good understanding of the underlying model of cell proliferation (i.e., the distribution of turnover rates in the population), one may obtain incorrect estimates of cellular turnover rates, even if the quality of the fit to the data is acceptably good. Therefore, when analyzing experimental data, one should aim at using several alternative models for fitting, and investigate whether estimates of kinetically important parameters, such as the average rate of cell turnover, are independent of the model used. There are two possible outcomes of such an analysis. First, fitting multiple models to data may yield similar estimates of the average rate of cell turnover. This would imply that the average turnover rate can be robustly estimated from the data, even though the precise model for cell kinetics cannot be determined from such an analysis. Second, the estimate of the average turnover rate may depend on the model that was used to fit the data, while the quality of the fit of various models to the data was similar. In this case, the estimate of the average rate of cell turnover is not robust to changes in the model. Additional data on cell kinetics (e.g., the fraction of cells in division or the fraction of dying cells) would then be required to discriminate between the alternative models for cell kinetics, and to obtain more confident estimates of the average rate of cell turnover.

### Fitting experimental data

We next sought to determine how well the new kinetic heterogeneity models fit experimental data. Using deuterated glucose, Mohri et al. [2] obtained labeling data of T lymphocytes from uninfected healthy human volunteers and from chronically HIV-infected patients. Previously, these data were fitted using an extended 4-parameter source model, to estimate the rates of cell division and death of T lymphocytes in healthy humans, and to obtain insights into how these rates change upon HIV-infection [2]. Lymphocytes were sorted into CD4^+^ and CD8^+^ T cells, without distinguishing between their naive and memory subpopulations. Since naive T cells have a much slower rate of turnover than memory T cells [3], it is natural to assume an asymptote in the fraction of labeled nucleotides of unsorted CD4^+^ and CD8^+^ T cells.

We have refitted the labeling data from the four healthy human volunteers studied by Mohri et al. [2], again using the three models for cell proliferation: the Asymptote model (eqn. (1)), the Exponential model (with a fraction 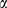 of cells with exponentially distributed turnover rates, eqn. (8)) and the Gamma model (with gamma distributed turnover rates, eqn. (4)). The data were fitted simultaneously for all four healthy volunteers while searching for the minimal number of parameters that describe the data with reasonable quality (using a partial F-test for nested models [15]). Because cells with deuterium-labeled DNA appear in circulation only a few days after the start of labeling [5], we allowed for a time delay in our model.

**Figure 6:**
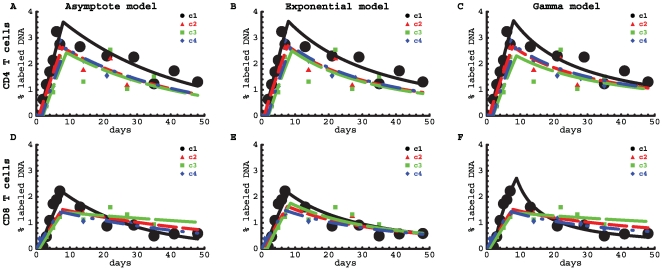
Fits of the deuterium labeling data with mathematica models. Data on labeling of CD4^+^ (top rows) and CD8^+^ (bottom rows) T cells in four healthy humans were fitted by three models: the Asymptote model (panels A and D), the Exponential model, in which a fraction 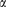 of cells have exponentially-distributed turnover rates (eqn. (8), panels B and E), and the Gamma model, with gamma-distributed turnover rates (eqn. (4), panels C and F). Experimental data obtained from Mohri et al. [2] are shown as symbols and the curves are the best model fits. The sum of squared residuals of the model fits to the data on the dynamics of CD4^+^ T cells are 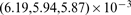 for the Asymptote model, the Exponential model and the Gamma model, respectively. The sum of squared residuals of the model fits to the data on the dynamics of CD8^+^ T cells are 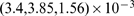 for the Asymptote model, the Exponential model and the Gamma model, respectively. Note that the two explicit kinetic heterogeneity models describe these data with similar (Exponential model) or even better (Gamma model) quality compared to the Asymptote model.

**Figure 7:**
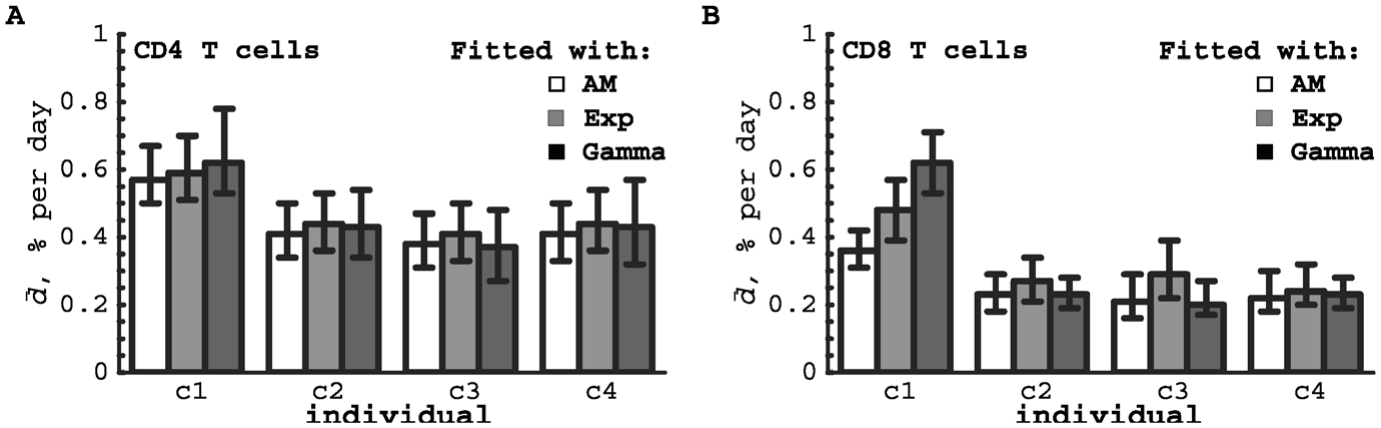
Estimates of the average turnover rates of human T cells. Data on deuterium labeling of CD4^+^ (panel A) and CD8^+^ (panel B) T cells in four healthy humans were fitted with three different models: the Asymptote model (empty bars), the Exponential model (gray bars), in which a fraction 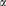 of the cells have exponentially-distributed turnover rates, and the Gamma model (black bars) with gamma-distributed turnover rates. Best fits of the data are shown in Figure 6, and estimates of all parameters of the models are shown in Table S2 and S3. Confidence intervals were obtained by bootstrapping the residuals with 1000 simulations. Note that all models deliver very similar estimates for the average turnover rate 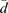, with the exception of the estimated CD8^+^ T-cell turnover rates in individual c1 which are highly model-dependent.

Overall, the models described the data reasonably well (Figure 6 and Table S2 and S3). For CD4^+^ T cells, the average turnover rate and the delay at which labeled cells appeared in the blood did not differ significantly between patients (Table S2). For all volunteers, the average rate of turnover was about 0.46% per day with a corresponding estimated half-life of 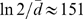 days. There was an average delay of one day before labeled cells appeared in the blood. The average turnover rate of CD4^+^ T cells from control c1 was always higher than that of the other individuals, irrespective of the model used (Figure 7A), which may be a sign of an immune response to an infection in c1 (see also below). Both the Asymptote model (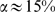) and the Exponential model (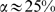) predicted an asymptote in labeling that is smaller than the fraction of memory phenotype CD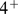 T cells in humans of that age [3]. The Gamma model could describe these data even better than the other two models with no need for an asymptote. The observation that the estimate of the asymptote can differ dramatically between different models reconfirms our statement that this parameter is of little use for data interpretation [11].

For CD8^+^ T cells, the parameters differed significantly between different healthy volunteers, with the exception of the asymptote level 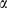 in the Exponential model which could be fixed between individuals. The estimates of the average turnover rates of CD8^+^ T cells in healthy volunteers c2–c4 did not strongly depend on the model that was used to fit the data. However, the estimated turnover rate of CD8^+^ T cells in individual c1, which was much higher than the estimated turnover rate in the other healthy volunteers, depended strongly on the model used and was estimated to be the highest when using the Gamma model. The latter model fitted the labeling data from all four individuals very well and reproduced the non-exponential change in the fraction of labeled DNA in the downlabeling phase (Figure 6F). In all four healthy individuals, CD8^+^ T cells turned over at a slower rate than CD4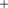 T cells; the average turnover rate of CD8^+^ T cells was 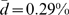 per day with a corresponding half-life of 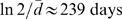. The fits of the Asymptote model and the Exponential model predicted an asymptote in labeling of 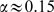 (Table S3). Even though the Gamma model lacks an explicit asymptote lower than 1, it fitted these data with equally good quality as the models with explicit asymptotes. Allowing for an explicit asymptote in the Gamma model did not improve the quality of the fit (CD4^+^ T cells: 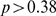, CD8^+^ T cells: 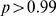, F-test), and the estimated average lymphocyte turnover rates were not affected by the addition of an explicit asymptote (results not shown).

It is important to investigate whether the good description of the data of the model with gamma distributed turnover rates is achieved with biologically reasonable parameter values. In all data we estimated the shape parameter of the gamma distribution to be small, i.e. 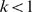, but 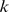 was estimated to be larger than 0.1 in seven of the eight fits. Low values of the shape parameter 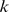 imply that in the population most cells turn over at very slow rates while a few populations turn over very rapidly. To investigate whether such a distribution is biologically reasonable, we calculated the fraction of cells in the population with a turnover rate higher than 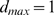 per day which is the maximal rate of CD8^+^ T-cell proliferation in rhesus macaques [16]. This fraction is given by 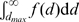 for the estimated parameters of the distribution (see Table S2 and S3). For most fits, the fraction of cells with turnover rates higher than 1 per day is 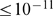, and given the estimated total number of lymphocytes in humans of 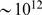 [17], that would yield only a few cells with unrealistically high rates of turnover. However, for the CD8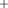 T cells of healthy volunteer c1 we found that 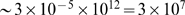 cells turn over at rates higher than 1 per day, which is unrealistically high.

To investigate this further we reanalyzed the CD8^+^ T-cell labeling data of individual c1 using several extended models. In the first model, a fraction 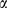 of cells in the population have gamma-distributed turnover rates while the other fraction (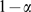) of cells turn over at the highest possible rate 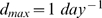. This situation may correspond to a scenario where a small fraction of CD8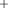 T cells is responding to an infection. However, this model failed to describe the data with biologically reasonable parameter values (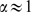 and 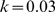).

In the second extended model, the gamma distribution of turnover rates was truncated at a maximal value 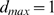 (see Text S1 for analytical results). The fit of this model to the labeling data for individual c1 was of similar quality as the fit in which the gamma distribution was not truncated, and it delivered similar estimates for the average turnover rate and the shape parameter (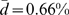 per day and 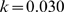, results not shown). We estimate that in healthy volunteer c1 about 0.1% of all CD8^+^ T cells are turning over rapidly at rates between 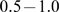 per day, which is reasonable. For example, in mice responding to lymphocytic choriomeningitis virus (LCMV) infection, at the peak of the immune response more than 50% of all CD8^+^ T cells in the spleen are specific for the virus [18],[19].

Finally, in the third model, we assumed that the CD8^+^ T-cell population in volunteer c1 consists of naive, memory and effector T-cell subpopulations with 3 different rates of turnover (see Eqn. (2) with 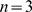). Assuming that the rate of turnover of naive T cells is 0 and that letting for effector cells 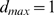 per day, we could obtain excellent fits of the labeling data with an estimated average turnover rate 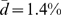 per day (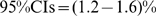 per day) which is much higher than estimates obtained by other models (Figure 7). Using model selection methods such as the Akaike Information Criterion, we found equal support for the latter model and the model in which the turnover rates follow a truncated gamma distribution [20, results not shown]. We can conclude, therefore, that the average turnover rate of CD8^+^ T cells in volunteer c1 is at least 0.62% per day (Gamma model) and could be as high as 1.4% per day (Three population model). In summary, it seems that the average turnover rate of both CD4^+^ and CD8^+^ T cells was increased in individual c1 as compared to other individuals, and this could be explained by a normal immune response in this otherwise healthy volunteer. Differences in CD8^+^ T cell kinetics between individuals c1 on the one hand and c2–c4 on the other, were in fact to be expected from visual inspection of the labeling data, because those from individual c1 reached a higher peak and had a faster decline in the fraction of labeled DNA after the peak than those of the other volunteers (see Figure 6F).

## Discussion

In this paper we have analyzed the models that are commonly used in the literature to estimate the rates of cell turnover from deuterium labeling data (see Table 1 for a summary of our main results). We have shown that the three most commonly used models are mathematically identical and therefore provide identical fits to the data. These models, however, differ in the biological interpretation of the estimated parameters [13]. The simplest summary of labeling data is provided by a model that has two parameters: 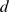 as the rate of cell death in the population, and 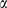 as the fraction of cells that undergo turnover, which determines the asymptote of the uplabeling phase (see eqn. (1)). In this model, 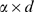 gives the estimated average rate of cell turnover. We have extended this model by allowing for multiple sub-populations 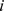 of size 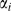 with different turnover rates 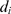 (see Eqn. (2)). This extended model can be used to investigate potential heterogeneity of cell populations, by fitting labeling data with a model that has one, two, or more sub-populations with different turnover rates. Using standard techniques of model selection (e.g., the partial F-test or the Akaike Information Criterion), one can investigate which of those models describes the labeling data best, given the number of model parameters [15],[20], or one can study whether the estimated average turnover rate is converging to an invariant value by increasing the number of compartments (work in progress).

**Table 1:** Summary of the major findings of the paper.

**What are the most important results of our analysis?**	
1.	Different models have previously been used to interpret deuterium-labeling data and have reached different conclusions [13]. We have shown that in terms of the average turnover rate all these previous models share an identical mathematical structure. Therefore, interpretation of the labeling data when expressed via the average turnover rate, should not depend on the model used to fit the data (given that the tested models assume identical distribution of turnover rates in the population, see also below).
2.	In contrast with previous approaches, our novel model predicts a non-exponential accumulation and decay of the fraction of labeled nucleotides during the labeling and delabeling phases.
3.	Our model naturally explains the observed fast loss of labeled DNA during the Figure 2 phase after a short labeling period, as compared to that after a long labeling period, due to preferential labeling of lymphocyte sub-populations with a rapid rate of turnover.
4.	From the dynamics of the fraction of labeled nucleotides, the new model estimates the distribution of turnover rates in the population, that is the fraction of cells that are turning over at a particular rate. This allows one to investigate how various conditions (e.g., infection, disease and treatment) affect the distribution of turnover rates in the population rather than just the mean.
**What are the implications of our results to the interpretation of labeling studies?**	
1.	The estimation of the average turnover rate of lymphocytes may depend on the distribution of turnover rates that one assumes for a population of cells. Therefore, the interpretation of experimental results may depend on the model used to analyze the data (given that different models assume different distributions of turnover rates in the population).
2.	Additional data on cell proliferation and/or cell death (e.g., short BrdU pulses, distribution of DNA, Ki67 or annexin V expression) will be helpful in determining which of the alternative models are most consistent with the data.
**How can labeling studies contribute to the general understanding of immunological processes?**	
1.	Using labeling of dividing cells with deuterium one can in principle determine the average rate of lymphocyte turnover and the distribution of turnover rates in the population. Comparing the average and the distribution of the rates of cell turnover between healthy individuals and various conditions (e.g., aged individuals, chronically infected, and transplantation patients) may help us to better understand these conditions.
2.	For example, for some cancers it is not known whether the growth of a population of cancer cells is mostly due to increased proliferation of cells, or largely due to decreased apoptosis. Determining the rates of cell turnover using deuterium labeling could help one to choose drugs that specifically target either proliferation or death of cancer cells [13].

For the case where the number of sub-populations is large, we derived a model with continuous kinetic heterogeneity. For several continuous distributions such as the exponential and the gamma distribution, the model predicts that the initial rate of loss of labeled DNA after label withdrawal is determined by the duration of the labeling period as has been observed experimentally [9]. Moreover, in the model the average turnover rate, which determines the initial rate of label accumulation in the population, turned out to be independent of the length of the labeling period. However, it should be noted that the average rate of cell turnover that is estimated from experimental data using, for example, the Asymptote model, may in fact depend on the duration of labeling [10, Den Braber et al. (in prepartion)]. Potential reasons for this discrepancy will be investigated in more detail elsewhere.

Previous models had certain artifacts: the asymptote labeling level was dependent on the length of the labeling period, and the accrual of labeled DNA during the uplabeling phase and the loss of labeled DNA during the downlabeling period were always described by single exponential functions. It is, therefore, unclear whether such limited models provide a good description of truly kinetically heterogeneous populations. We have shown that deuterium labeling data could be fitted and parameters estimated reliably, using a model that assumes a large number of kinetically heterogeneous subpopulations. By fitting artificial labeling data, we have validated these new models: they generally give good fits to the data and converge on average turnover rates that are close to the known average turnover rate. Moreover, the new explicit heterogeneity model outperformed the Asymptote model when it came to fitting experimental data, especially when the rates of label accumulation and loss are not exponential (see Figure 6). Importantly, due to its relatively general structure, all results of the kinetic heterogeneity model are applicable to both non-proliferating and proliferating lymphocytes, all having a distribution of turnover values (results not shown). Moreover, because the model naturally incorporates the dependence of the rate of label loss on the length of the labeling period, this is the first model that can be strictly applied to fit labeling data with different labeling periods.

We have focused our analysis on a particular type of kinetic heterogeneity in which kinetic properties of cells of a given subpopulation do not change over time and there is no exchange of cells between different sub-populations. Although we have not specified the nature of sub-populations, one possibility would be that cells within a sub-population share the same antigenic specificity (i.e., they are T-cell clones). In that case, within each functional compartment, and averaged over potential temporal heterogeneity, cells expressing the same antigen receptor would be assumed to have similar kinetic properties. It would be interesting to investigate whether T-cell clones or e.g. polyclonal T -cell populations sharing a particular phenotype (like CD44 or CD62L) are indeed kinetically sufficiently homogeneous to qualify as sub-populations of cells with similar kinetic properties.

However, during acute immune responses, the assumption of constant kinetic properties of all cells in a sub-population may be violated. Over the course of an infection, lymphocytes do change their kinetic properties over time (e.g., [21]). Under such circumstances one should take such a type of *temporal* heterogeneity into account. This requires future work to develop sufficiently simple models from earlier examples [22],[23]. Generally, future studies should aim at testing multiple models in how well they describe the labeling data and whether these models deliver similar estimates of important kinetic parameters such as the average rate of cell turnover.

## Methods

When fitting experimental data, the models were extended to allow for the initial delay in the labeling of cells (see also [2]). For example, including a delay in the Asymptote model (given by eqn. (1)) takes the form
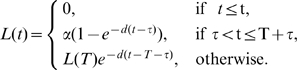
(9)


To normalize the residuals of the model fits to experimental data, given that the data are expressed as proportions, the data and the model predictions were transformed as 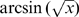 where 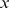 is the frequency of labeled DNA in the population [24]. The models were fitted according to the least squares method by using the FindMinimum routine in Mathematica. Confidence intervals were calculated by bootstrapping the residuals with 1000 simulations.

## Supporting Information

Table S1 Estimates of the parameters after fitting three models to three sets of artificial data.
(0.06 MB PDF)

Table S2 Average turnover rates of CD4+ T cells from four healthy humans as estimated by fitting experimental data.
(0.06 MB PDF)

Table S3 Average turnover rates of CD8+ T cells from four healthy humans as estimated by fitting experimental data.
(0.05 MB PDF)

Text S1 Derivation of the model with continuous kinetic heterogeneity.
(0.09 MB PDF)
