# Supplementary material for: Explicit Kinetic Heterogeneity: Mathematical Models for Interpretation of Deuterium Labeling of Heterogeneous Cell Populations
Source: PLoS Comput Biol. 2010 Feb 5;6(2):e1000666. doi: 10.1371/journal.pcbi.1000666 (PMC2816685; doi:10.1371/journal.pcbi.1000666)
Supplement: Table S1 — Estimates of the parameters after fitting three models to three sets of artificial data. (0.06 MB PDF) [file pcbi.1000666.s001.pdf]

Data fitted with:		Data generated using: Gamma model		Exponential model		Two-populations model	
		7 days	15 days	7 days	15 days	7 days	15 days
Ass	$\bar{d}$	0.076 (0.068—0.085)	0.068 (0.06—0.077)	0.082 (0.073—0.096)	0.08 (0.07—0.092)	0.04 (0.034—0.046)	0.039 (0.032—0.047)
	d	0.121 (0.105—0.14)	0.117 (0.099—0.137)	0.203 (0.17—0.238)	0.199 (0.169—0.231)	0.052 (0.04—0.066)	0.054 (0.038—0.071)
Exp	$\bar{d}$	0.09 (0.082—0.104)	0.09 (0.076—0.1)	0.11 (0.105—0.122)	0.12 (0.104—0.137)	0.05 (0.042—0.055)	0.05 (0.040—0.055)
	α	0.83 (0.773—0.908)	0.76 (0.70—0.82)	0.51 (0.488—0.536)	0.48 (0.456—0.51)	1 (0.857—1.0)	1 (0.835–1.0)
Gam	$\bar{d}$	0.097 (0.085—0.112)	0.099 (0.085—0.117)	0.154 (0.14—0.169)	0.197 (0.152—0.271)	0.044 (0.038—0.052)	0.044 (0.036—0.054)
	k	0.594 (0.478—0.768)	0.44 (0.367—0.536)	0.197 (0.184—0.211)	0.159 (0.134—0.183)	1.329 (0.796—2.947)	1.132 (0.679—2.901)

Table S1: Estimates of the parameters after fitting three models to three sets of artificial data. Rows correspond to different models used to fit the data, and columns correspond to the models used to generate the data. We show parameter estimates of the Asymptote model (in which $\bar{d}$ expresses the average turnover rate of the fraction of cells undergoing turnover), the Exponential model (in which a fraction α of cells have exponentially distributed turnover rates), and the Gamma model (with gamma distributed turnover rates). Data were generated using the Gamma model (eqn. (4)), the Exponential model (eqn. (8)), and the Two-populations model (eqn. (2)). The 95% confidence intervals, that are shown next to the mean values, were obtained by bootstrapping the residuals with 1000 simulations. Data have been generated for two labeling periods, of 7 and 15 days, respectively. For all data, the average rate of turnover was fixed at 0.1/day. Other parameters used to generate the data are: $k = 0.5$ (Gamma model), $\alpha = 0.5$ and $\bar{d}_\alpha = 0.2$/day (Exponential model), $d_1 = 1$/day, and $\alpha = 0.07$ (Two-populations model).
