# Supplementary material for: Explicit Kinetic Heterogeneity: Mathematical Models for Interpretation of Deuterium Labeling of Heterogeneous Cell Populations
Source: PLoS Comput Biol. 2010 Feb 5;6(2):e1000666. doi: 10.1371/journal.pcbi.1000666 (PMC2816685; doi:10.1371/journal.pcbi.1000666)
Supplement: Table S2 — Average turnover rates of CD4+ T cells from four healthy humans as estimated by fitting experimental data. (0.06 MB PDF) [file pcbi.1000666.s002.pdf]

| | **Data fitted with:** | | |
|---|---|---|---|
| | Asymptote model | Exponential model | Gamma model |
| $d/\bar{d}_\alpha/k$ | 2.87 (2.22—3.65) | 1.94 (1.46—2.6) | 0.12 (0.09—0.17) |
| $\bar{d}_1$, % $day^{-1}$ | 0.57 (0.5—0.67) | 0.59 (0.51—0.7) | 0.62(0.53—0.78) |
| $\bar{d}_2$ | 0.41 (0.34—0.5) | 0.44 (0.36—0.53) | 0.43 (0.34—0.54) |
| $\bar{d}_3$ | 0.38 (0.31—0.47) | 0.41 (0.33—0.5) | 0.37 (0.27—0.48) |
| $\bar{d}_4$ | 0.41 (0.33—0.5) | 0.44 (0.36—0.54) | 0.43 (0.32—0.57) |
| $\tau_1$, *day* | 1. (0.91—1.51) | 1. (0.93—1.53) | 1. (0.93—1.57) |
| $\tau_2$ | 0.78 (0.28—1.) | 0.81 (0.35—1.) | 0.8 (0.31—1.) |
| $\tau_3$ | 1.97 (1.—2.65) | 2.06 (1.14—2.7) | 1.87 (0.73—2.67) |
| $\tau_4$ | 1.7 (0.98—2.45) | 1.83 (1.—2.54) | 1.79 (1.—2.58) |
| RSS, $10^{-3}$ | 6.19 | 5.94 | 5.87 |

**Table S2:** Average turnover rates of $CD4^+$ T cells from four healthy humans as estimated by fitting the data from Mohri et al. [2] using the Asymptote model, the Exponential model, and the Gamma model. The best fits of the models resulted in different average rates of cell turnover $\bar{d}_i$ and initial delays of labeling $\tau_i$. Other parameters, that could be assumed to be identical between different individuals, are the death rate of labeled cells $d$ (Asymptote model), the rate of turnover $\bar{d}_\alpha$ of the turning-over sub-population in the Exponential model, and the shape parameter $k$ in the model with gamma distributed turnover rates. For the model with gamma distributed turnover rates, an asymptote level $\alpha = 1$ provided the best fit of the data. The quality of the fit is illustrated by the residual sum of squares (RSS). The 95% confidence intervals were obtained by bootstrapping the residuals with 1000 simulations.
