# Supplementary material for: Explicit Kinetic Heterogeneity: Mathematical Models for Interpretation of Deuterium Labeling of Heterogeneous Cell Populations
Source: PLoS Comput Biol. 2010 Feb 5;6(2):e1000666. doi: 10.1371/journal.pcbi.1000666 (PMC2816685; doi:10.1371/journal.pcbi.1000666)
Supplement: Table S3 — Average turnover rates of CD8+ T cells from four healthy humans as estimated by fitting experimental data. (0.05 MB PDF) [file pcbi.1000666.s003.pdf]

	Data fitted with:		
	Asymptote model	Exponential model	Gamma model
$\alpha_1/\alpha/k_1$	0.08 (0.07—0.10)	0.13 (0.12—0.16)	0.033 (0.028—0.039)
$\alpha_2/\alpha/k_2$	0.13 (0.09—0.73)	0.13 (0.12—0.16)	0.116 (0.066—0.341)
$\alpha_3/\alpha/k_3$	0.26 (0.10—1.0)	0.13 (0.12—0.16)	0.347 (0.099—10^7)
$\alpha_4/\alpha/k_4$	0.10 (0.07—0.28)	0.13 (0.12—0.16)	0.082 (0.05—0.155)
$\bar{d}_1$, % per day	0.36 (0.31—0.43)	0.48 (0.39—0.57)	0.62 (0.53—0.71)
$\bar{d}_2$	0.23 (0.18—0.30)	0.27 (0.21—0.34)	0.23 (0.19—0.28)
$\bar{d}_3$	0.21 (0.17—0.31)	0.29 (0.22—0.39)	0.2 (0.17—0.27)
$\bar{d}_4$	0.22 (0.18—0.3)	0.24 (0.2—0.32)	0.23 (0.19—0.28)
τ_1	0.99 (0.73—1.38)	1.76 (1.33—1.95)	1.89 (1.75—1.98)
τ_2	0.65 (0.—0.94)	0.76 (0.11—0.99)	0.65 (0.28—0.92)
τ_3	0.85 (0.—1.99)	1.73 (0.59—2.4)	0.82 (0.—1.81)
τ_4	0. (0.—0.63)	0. (0.—0.64)	0. (0.—0.55)
RSS, 10^{-3}	3.4	3.85	1.56

Table S3: Average turnover rates of $CD8^+$ T cells from four healthy humans as estimated by fitting the data from Mohri et al. [2] using the Asymptote model, the Exponential model and the Gamma model. The best fits of the models resulted in different parameter estimates for all volunteers, with the exception of the fraction of turning over cells α in the Exponential model (which was fitted as one parameter for all individuals). As for $CD4^+$ T cells, in the model with gamma distributed turnover rates, the asymptote level $\alpha = 1$ provided the best fit of the data. The shown 95% confidence intervals were obtained by bootstrapping the residuals with 1000 simulations.
