# Supplementary material for: Explicit Kinetic Heterogeneity: Mathematical Models for Interpretation of Deuterium Labeling of Heterogeneous Cell Populations
Source: PLoS Comput Biol. 2010 Feb 5;6(2):e1000666. doi: 10.1371/journal.pcbi.1000666 (PMC2816685; doi:10.1371/journal.pcbi.1000666)
Supplement: Text S1 — Derivation of the model with continuous kinetic heterogeneity. (0.09 MB PDF) [file pcbi.1000666.s004.pdf]

Text S1

Derivation of the model with continuous kinetic heterogeneity

The average label incorporation in a population consisting of n sub-populations is given by eqn. (2). If the number of sub-populations is large ($n \to \infty$), one could switch from summation to integration in eqn. (2). If α_i is the fraction of cells in sub-population i with turnover rate d_i, then, as $n \to \infty$, $\alpha_i = f(d)\mathrm{d}d$, where the latter is the probability that a randomly chosen cell from a population will have a turnover rate in the range $(d, d + \mathrm{d}d)$. If $\sum_{i=1}^{n} \alpha_i = 1$, then $f(d)$ is also normalized to 1. Then for $t > T$, eqn. (2) can be rewritten in a different form

$$L(t) = \lim_{n\to\infty} \left[1 - \sum_{i=1}^{n} \alpha_i \mathrm{e}^{-d_i t} \right] = 1 - \int_0^\infty f(d)\mathrm{e}^{-dt}\mathrm{d}d. \tag{S.1}$$

Similarly, from eqn. (2), during the delabeling period, the fraction of labeled DNA is given by

$$L(t) = \int_0^\infty f(d)\mathrm{e}^{-d(t-T)}\mathrm{d}d - \int_0^\infty f(d)\mathrm{e}^{-dt}\mathrm{d}d. \tag{S.2}$$

One can then calculate several important characteristics that determine the change of the fraction of labeled DNA over time. First, the initial uplabeling rate can be calculated using eqn. (S.1) for small t, yielding

$$L(t) = 1 - \int_0^\infty f(d)\mathrm{e}^{-dt}\mathrm{d}d \approx 1 - \int_0^\infty f(d)(1 - dt)\mathrm{d}d = \bar{d}t. \tag{S.3}$$

where $\bar{d} = \int_0^\infty d f(d)\mathrm{d}d$, and $\int_0^\infty f(d)\mathrm{d}d = 1$ by definition. The importance of this result is that it demonstrates that for any distribution $f(d)$, the estimated initial rate of uplabeling is determined only by the average rate of cell turnover.

During delabeling, the initial change in the fraction of labeled DNA (for $t = T + \varepsilon$ with ε relatively small), can be calculated from:

$$L(t) \;\; = \;\; \int_0^\infty f(d)\mathrm{e}^{-d(t-T)}\mathrm{d}d - \int_0^\infty f(d)\mathrm{e}^{-dt}\mathrm{d}d = \int_0^\infty f(d)(1 - d\varepsilon)\mathrm{d}d -$$

$$\int_0^\infty f(d)\mathrm{e}^{-dT}(1-d\varepsilon)\mathrm{d}d = 1-\int_0^\infty f(d)\mathrm{e}^{-dT}\mathrm{d}d - \varepsilon\left[\bar{d}-\int_0^\infty df(d)\mathrm{e}^{-dT}\mathrm{d}d\right] =$$
$$L(T)-\varepsilon L(T)\left[\frac{\bar{d}-\int_0^\infty df(d)\mathrm{e}^{-dT}\mathrm{d}d}{1-\int_0^\infty f(d)\mathrm{e}^{-dT}\mathrm{d}d}\right], \qquad \text{(S.4)}$$

where $L(T) = 1-\int_0^\infty f(d)\mathrm{e}^{-dT}\mathrm{d}d$. Then the initial per capita rate of loss of labeled DNA, d^*, can be calculated for short and long labeling periods. For short labeling periods $T \to 0$, and we find

$$d^* = \frac{\bar{d}-\int_0^\infty df(d)\mathrm{e}^{-dT}\mathrm{d}d}{1-\int_0^\infty f(d)\mathrm{e}^{-dT}\mathrm{d}d} = \frac{\bar{d}-\int_0^\infty df(d)(1-dT)\mathrm{d}d}{1-\int_0^\infty f(d)(1-dT)\mathrm{d}d} = \frac{\overline{d^2}}{\bar{d}} = \bar{d}+\frac{\mathrm{var}(d)}{\bar{d}}, \qquad \text{(S.5)}$$

where $\mathrm{var}(d) = \overline{d^2}-(\bar{d})^2$ is the variance of the turnover rates in the population. When the labeling period is long, $T \to \infty$, the per capita rate of label loss is

$$d^* = \frac{\bar{d}-\int_0^\infty df(d)\mathrm{e}^{-dT}\mathrm{d}d}{1-\int_0^\infty f(d)\mathrm{e}^{-dT}\mathrm{d}d} = \bar{d}, \qquad \text{(S.6)}$$

since all terms $\mathrm{e}^{-dT} \to 0$ as $T \to \infty$. This confirms the conjecture of Asquith et al. [9] that d^* should approach the average rate of turnover after long labeling period. We now add that the maximal difference between the average turnover rate, $\bar{d}$, and the loss rate of labeled cells, d^*, is set by the distribution of turnover rates in the population.

Particular solutions of the kinetic heterogeneity model

For several simple distributions of the turnover rates, we can obtain analytical solutions for the change in the fraction of labeled DNA during the labeling experiment.

Exponential distribution. In this case $f(d) = (1/\bar{d})\mathrm{e}^{-d/\bar{d}}$, where $\bar{d}$ is the average turnover rate in the population. Using eqn. (S.1) and (S.2), we find

$$L(t) = 1-\frac{1}{\bar{d}}\int_0^\infty \mathrm{e}^{-dt-d/\bar{d}}\mathrm{d}d = 1-\frac{1}{(t+1/\bar{d})\bar{d}} = \frac{\bar{d}t}{1+\bar{d}t}, \quad t \le T, \qquad \text{(S.7)}$$

$$
\begin{aligned}
L(t) &= \frac{1}{\bar{d}}\int_0^\infty \mathrm{e}^{-d(t-T)-d/\bar{d}}\mathrm{d}d - \frac{1}{\bar{d}}\int_0^\infty \mathrm{e}^{-dt-d/\bar{d}}\mathrm{d}d = \frac{1}{1+\bar{d}(t-T)} - \frac{1}{1+\bar{d}t} \\
&= L(T)\frac{1+\bar{d}T}{(1+\bar{d}t)(1+\bar{d}(t-T))}, \quad t > T. \qquad \text{(S.8)}
\end{aligned}
$$

When $\bar{d}t \ll 1$, the fraction of labeled DNA is simply $L(t) \approx \bar{d}t$, i.e., the initial rate of increase is again given by the average turnover rate $\bar{d}$. The initial rate of decline during delabeling is less clear. Let us define $t = T + \varepsilon$ where $\bar{p}\varepsilon \ll 1$. Then after cessation of label administration, using Taylor's expansion we find

$$
\begin{aligned}
L(t) &= \frac{1}{1+\bar{d}\varepsilon} - \frac{1}{1+\bar{d}T+\bar{d}\varepsilon} = 1 - \bar{d}\varepsilon - \frac{1}{1+\bar{d}T} + \frac{\bar{d}\varepsilon}{(1+\bar{d}T)^2} + o(\varepsilon) = \\
&= L(T) - L(T)\bar{d}\left(1 + \frac{1}{1+\bar{d}T}\right)\varepsilon + o(\varepsilon) \qquad \text{(S.9)}
\end{aligned}
$$

where $L(T) = 1 - 1/(1+\bar{d}T)$. This expression shows that the initial per capita rate of loss of labeled DNA, $d^* = \bar{d}\left(1 + \frac{1}{1+\bar{d}T}\right)$, decreases with increasing length of the labeling period [9]. If $\bar{d}T \ll 1$ (short labeling), the initial per capita decay rate is $d^* \approx 2\bar{d}$.

Gamma distribution. In this case $f(d) = \lambda(\lambda d)^{k-1}\mathrm{e}^{-\lambda d}/(k-1)!$, where λ and k are the scale and shape parameters, respectively, and the average rate of cell turnover $\bar{d} = k/\lambda$, $\sigma_d^2 = \bar{d}^2/k$, and $CV = \sigma_d/\bar{d} = 1/\sqrt{k}$. Note that when $k = 1$, the gamma distribution is identical to the exponential distribution. Substituting the gamma distribution in eqn. (S.1), we find

$$
\begin{aligned}
L(t) &= 1 - \frac{\lambda^k}{(k-1)!}\int_0^\infty d^{k-1}\mathrm{e}^{-dt-\lambda d}\mathrm{d}d \\
&= 1 - \frac{\lambda^k}{(k-1)!(t+\lambda)^k}\int_0^\infty d^{k-1}(t+\lambda)^k\mathrm{e}^{-d(t+\lambda)}\mathrm{d}d \\
&= 1 - \frac{\lambda^k}{(t+\lambda)^k} = 1 - \left[1 + \frac{\bar{d}t}{k}\right]^{-k}, \quad t \le T. \qquad \text{(S.10)}
\end{aligned}
$$

Using eqn. (S.2) and proceeding similarly, we find the change in the fraction of labeled DNA during delabeling:

$$
L(t) = \left[1 + \frac{\bar{d}(t-T)}{k}\right]^{-k} - \left[1 + \frac{\bar{d}t}{k}\right]^{-k}, \quad t > T. \qquad \text{(S.11)}
$$

(S.12)

The initial rate of increase in the fraction of labeled cells is also independent of the length of the labeling period and, initially, for $t = \varepsilon$ (such as $\bar{d}\varepsilon \ll 1$) is

$$L(\varepsilon) = 1 - \left[\frac{1}{1+\frac{\bar{d}\varepsilon}{k}}\right]^k = 1 - (1 - k\bar{d}\varepsilon/k) + o(\varepsilon) = \bar{d}\varepsilon + o(\varepsilon). \tag{S.13}$$

as expected. The initial per capita rate of loss of labeled DNA is somewhat more complex. For times $t = T + \varepsilon$ such as $\bar{d}\varepsilon \ll 1$, using Taylor's expansion, we find

$$L(t) = \left[1+\frac{\bar{d}\varepsilon}{k}\right]^{-k} - \left[1+\frac{\bar{d}(T+\varepsilon)}{k}\right]^{-k} = 1 - \frac{1}{\left(1+\frac{\bar{d}T}{k}\right)^k} - \bar{d}\varepsilon\left[1 - \frac{1}{\left(1+\frac{\bar{d}T}{k}\right)^k}\right]. \tag{S.14}$$

It is useful to rewrite this expression in terms of $L(T)$:

$$L(t) = L(T) - L(T)\frac{\bar{d}\varepsilon}{1+\bar{d}T/k}\left[\frac{(1+\bar{d}T/k)^{k+1}-1}{(1+\bar{d}T/k)^k - 1}\right] = L(T)(1 - d^*\varepsilon). \tag{S.15}$$

where $d^* = \frac{\bar{d}}{1+\bar{d}T/k}\left[\frac{(1+\bar{d}T/k)^{k+1}-1}{(1+\bar{d}T/k)^k-1}\right]$ is the initial per capita loss of labeled DNA. For short labeling ($\bar{d}T \ll 1$), the initial per capita decay rate is $d^* \approx \bar{d}(k+1)/k$, and as the shape parameter k becomes larger, the decline rate d^* approaches the average proliferation rate $\bar{d}$. For long labeling periods ($\bar{d}T \gg 1$), $d^* \approx \bar{d}$, as expected.

Truncated gamma distribution. Under some circumstances the distribution of turnover rates may allow for too high rates of cell turnover. To circumvent this problem one may use a truncated distribution. For a gamma distribution truncated at maximal value d_{max}, the distribution is similar as above with an added normalization constant C, $f(d) = C^{-1}\lambda(\lambda d)^{k-1}\mathrm{e}^{-\lambda d}/(k-1)!$. The constant C is found by normalizing the probability distribution

$$C = \int_0^{d_{max}} f(d)\mathrm{d}d = 1 - \frac{\Gamma(k, d_{max}\lambda)}{(k-1)!}, \tag{S.16}$$

where $\Gamma(k,d) = \int_d^\infty x^{k-1}e^{-x}\mathrm{d}x$ is an incomplete gamma function. The average turnover rate then has to be calculated numerically

$$\bar{d} = C^{-1}\int_0^{d_{max}} df(d)\mathrm{d}d = \frac{k}{\lambda}\left[\frac{1-\frac{\Gamma(k+1,d_{max}\lambda)}{k!}}{1-\frac{\Gamma(k,d_{max}\lambda)}{(k-1)!}}\right]. \tag{S.17}$$

For the fraction of labeled nucleotides, we proceed as in eqn. (S.10) and obtain

$$\begin{aligned} L(t) &= 1-\frac{\lambda^k}{C(k-1)!}\int_0^{d_{max}} d^{k-1}\mathrm{e}^{-dt-\lambda d}\mathrm{d}d \\ &= 1-\frac{\lambda^k}{C(k-1)!(t+\lambda)^k}\int_0^{d_{max}(t+\lambda)} x^{k-1}\mathrm{e}^{-x}\mathrm{d}x \\ &= 1-\frac{\lambda^k}{C(k-1)!(t+\lambda)^k}\left[(k-1)!-\int_{d_{max}(t+\lambda)}^\infty x^{k-1}\mathrm{e}^{-x}\mathrm{d}x\right] \\ &= 1-\frac{\lambda^k}{(t+\lambda)^k}\times\frac{(k-1)!-\Gamma(k,d_{max}(\lambda+t))}{(k-1)!-\Gamma(k,d_{max}\lambda)}, \quad t\le T. \end{aligned} \tag{S.18}$$

During delabeling ($t > T$), we proceed as in eqn. (S.11) and find

$$\begin{aligned} L(t) = &\frac{\lambda^k}{(\lambda+t-T)^k}\times\frac{(k-1)!-\Gamma(k,d_{max}(\lambda+t-T))}{(k-1)!-\Gamma(k,d_{max}\lambda)} - \\ &\frac{\lambda^k}{(t+\lambda)^k}\times\frac{(k-1)!-\Gamma(k,d_{max}(\lambda+t))}{(k-1)!-\Gamma(k,d_{max}\lambda)}. \end{aligned} \tag{S.19}$$

Since $\lim_{d_{max}\to\infty}\Gamma(k,d_{max}) = 0$, at $d_{max}\to\infty$,

the fraction of labeled nucleotides becomes identical to eqn. (4) with $\lambda = k/\bar{d}$.

Non-parametric estimates of the distribution of proliferation rates in the population

Mathematically, the last term in eqn. (S.1) is a Laplace transformation of $f(p)$. This result stems from the assumption of exponentially distributed inter-division times of cells and raises

the intriguing possibility that from the change in the fraction of labeled DNA during label administration, one can estimate the distribution of proliferation rates in the population. Let us denote $f^*(t)$ as the Laplace transformation of $f(d)$:

$$f^*(t) = \mathcal{L}[f(d)] = \int_0^\infty f(d)\mathrm{e}^{-dt}\mathrm{d}d. \tag{S.20}$$

From eqn. (S.1), one finds the distribution of proliferation rates in the population using the inverse Laplace transformation:

$$f(d) = \mathcal{L}^{-1}[1 - L(t)]. \tag{S.21}$$

where $L(t)$ is a curve describing the change of the fraction of labeled DNA during label administration. Such a curve could be obtained in several ways, for example, by interpolating the data. Application of this method for the analysis of experimental data will be published elsewhere.
